# Recombinant growth hormone therapy in children with short stature in Kuwait: a cross-sectional study of use and treatment outcomes

**DOI:** 10.1186/s12902-015-0073-7

**Published:** 2015-12-03

**Authors:** Dalia Al-Abdulrazzaq, Abdullah Al-Taiar, Kholoud Hassan, Iman Al-Basari

**Affiliations:** Department of Pediatrics, Faculty of Medicine, Kuwait University, PO Box 24923, Safat, 13110 Kuwait; Department of Community Medicine, Faculty of Medicine, Kuwait University, PO Box 24923, Safat, 13110 Kuwait; Department of Pediatrics, Mubarak Al-Kabeer Hospital, Ministry of Health, Safat, Kuwait

**Keywords:** Short Stature, Growth Hormone, Therapy, Kuwait, Middle East

## Abstract

**Background:**

Recombinant Growth hormone (rGH) therapy is approved in many countries for treatment of short stature in a number of childhood diagnoses. Despite the increasing body of international literature on rGH use, there is paucity of data on rGH use in Kuwait and the broader Middle-East which share unique ethnic and socio-cultural backgrounds. This study aimed to describe the pattern of use and treatment outcomes of rGH therapy in Kuwait.

**Methods:**

This is a cross-sectional retrospective review of children treated with rGH in the Department of Pediatrics, in a major hospital in Kuwait between December 2013 and December 2014. Data were extracted using standard data extraction form and the response to rGH therapy was defined as a gain of ≥ 0.3 standard deviation score (SDS) of height per year.

**Results:**

A total of 60 children were treated with rGH in the center. Their Median (Interquartile) age at rGH initiation was 9.0 (6.2, 10.7) years. The most common indications for rGH therapy were Growth Hormone Deficiency (GHD) 23 (38.3 %), Idiopathic Short Stature (ISS) 12 (20.0 %) and Small for Gestational Age (SGA) 9 (15.0 %). After excluding patients with TS, no significant differences were found in gender of those who received rGH therapy in all indications combined or in each group (*p* ≥ 0.40). At 1-year follow-up, children in all groups had median height SDS change of ≥ 0.3 SDS except for children with ISS. Age at rGH initiation was negatively associated with 1-year treatment response, Adjusted odds ratio (AOR) 0.56 (95 % CI: 0.04–1.49); *p* = 0.011).

**Conclusions:**

GHD is the most common indication of rGH therapy. All indications except for ISS showed significant 1-year treatment response to therapy. Treatment outcomes in patients with ISS should be further investigated in Kuwait. Younger age at initiation of rGH therapy was independently associated with significant response to therapy suggesting the importance of identifying children with short stature and prompt initiation of rGH therapy.

## Background

Recombinant Growth Hormone (rGH) has been used to promote linear growth since 1985. rGH acts mainly through increasing the secretion of insulin-like growth factor-1 (IGF-1) [1]. The indications for rGH have gradually been extended from replacement therapy in Growth Hormone Deficiency (GHD) to an increasing number of conditions in which short stature is not due to GHD. The new indications include Small for Gestational Age (SGA), Idiopathic Short Stature (ISS) and Turner Syndrome (TS) in addition to other indications [1–3]. Since rGH was introduced, there have been several observational studies that described its effectiveness among children [4–7]. In most of the indications listed above, rGH therapy has been shown to improve final adult height [5, 7–9]. Despite the increasing body of international literature on rGH use, there is paucity of data on rGH use in Kuwait and the broader Middle-East which share unique ethnic and socio-cultural backgrounds.

Although the use of rGH therapy was limited when it was gradually introduced in 2001, the use of rGH therapy has recently increased with the increasing number of trained pediatric endocrinologists in the country. The cost of treatment is covered by the government for Kuwaiti nationals and by a local Patient-Aid Organization for non-Kuwaiti nationals. In Kuwait, as in many other countries in the Gulf region, the pattern of use of rGH therapy and the treatment outcomes remain mostly unknown. It is possible that the treatment outcome differs between different ethnicities and extrapolation of data from other settings is not always appropriate. Furthermore, description of the pattern of use of rGH therapy and the treatment outcomes in Kuwait and comparing these to international practices and treatment outcomes, are critical for the healthcare system of Kuwait which will help improve quality of care and resource utilization. In this study, we aimed to describe the pattern of use of rGH in a pediatric endocrine center in Kuwait and evaluate the 1-year response to therapy. We aimed also to identify the factors associated with significant treatment response.

## Methods

This is a cross-sectional retrospective review of children treated with rGH in the Department of Pediatrics, Mubarak Al-Kabeer Hospital, in Kuwait. These children were under follow-up at the Endocrine Clinic between December 2013 and December 2014. The Department of Pediatrics at this hospital is one of four departments which treat children with short stature. The department has a large coverage area in the country and serves a total pediatric population of approximately 250,000 [10]. The study was approved by the ethics committee of The Health Sciences Centre, Kuwait University and the ethics committee at The Ministry of Health in Kuwait.

Children and adolescents treated with rGH were identified using pharmacy prescription records at the Hospital. They were categorized according to indication of therapy to five groups namely: Growth Hormone Deficiency (GHD), Idiopathic Short Stature (ISS), Small for Gestational Age (SGA), Turner Syndrome (TS) and variants, and other indications (e.g. syndromic short stature, chronic renal disease and inflammatory bowel disease). Growth hormone deficiency was diagnosed based on two stimulation tests with clonidine and glucagon. A peak GH concentration after the stimulation of < 20 mIU/L (approximately equivalent to 7.5 ng/dL) was considered diagnostic of GHD. ISS was defined by a height below – 2 SDS without any findings of underlying disease as evident by a complete evaluation by a pediatric endocrinologist including stimulated GH levels [11]. SGA was diagnosed when birth weight is less than the 10^th^centile for gestational age [12]. rGH therapy was initiated at a dose of 25–30 μg/kg/day for children with GHD [2], 50 μg/kg/day for children with ISS [11], 35 μg/kg/day for children with SGA [13], and 50 μg/kg/day for children with TS and chronic renal disease [2].

The medical records of identified patients were reviewed and data were extracted using standard data extraction form. The data included baseline information at rGH initiation such as demographic data, indication of rGH therapy, anthropometric measures (weight, height, and body mass index [BMI]) expressed as standard deviation scores (SDS) determined by the World Health Organization child growth standards that are approved for use in Kuwait by the Ministry of Health [14]. Data were also extracted on pubertal status of each patient which was documented according to the Tanner stage. Pre-pubertal status was defined as Tanner stage 1 breast development for girls and testicular volume of < 4 mL for boys. For patients with missing data on pubertal status in their medical records, girls younger than the age of 8 years and boys younger than the age of 9 years at initiation of rGH were considered pre-pubertal. Data at 1-year follow-up were also extracted from medical records. These include anthropometric measures as above; and a significant response to rGH therapy at follow-up was defined as a gain of ≥ 0.3 SDS of height per year as per Ranke and Lindberg [15]. Pre-pubertal status at 1-year follow-up was defined as above; and missing data on pubertal status were also handled as above.

Statistical analysis was performed using STATA software13.1. Differences with p-value of less than 0.05 were deemed to be statistically significant. Continuous variables were expressed as mean (SD) when normally distributed or median (interquartile range: IQR) otherwise. Kruskal–Wallis test or ANOVA was used to test for the differences in continuous variables as appropriate; while Fisher’s exact test was used to test for differences in categorical variables. We also tested the differences in the distribution of gender in each treatment group using exact binomial probability test. Multiple logistic regression was used to investigate the baseline factors associated with significant response to rGH therapy at 1-year follow-up.

## Results

**Fig. 1:**
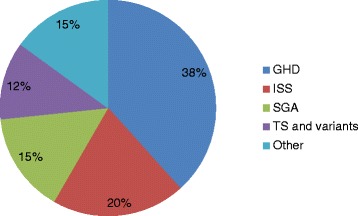
Indications of rGH Therapy in Kuwait. rGH: Recombinant Growth Hormone ; GHD: Growth Hormone Deficiency; ISS: Idiopathic Short Stature; SGA: Small for Gestational Age; TS: Turner Syndrome

Sixty patients were treated with rGH during the study period of whom 31 (51.7 %) were males. After excluding patients with TS from the analysis, no significant difference was found in gender of those who received rGH therapy in all indications combined or in each group (*p* ≥ 0.40) although only 9 out of 23 patients with GHD were females (*p* = 0.40). Moreover, there was no significant difference in baseline height SDS between males and females (all indications *p* = 0.90). Of the sixty patients, 44 (73.3 %) were Kuwaitis; and the most common indication for therapy was GHD (Fig. 1). The baseline characteristics of all the patients according to indication of therapy are summarized in Table 1. There were no significant differences between the treated groups in relation to age, nationality, pubertal status, height SDS, and BMI SDS at the initiation of rGH therapy.

**Table 1:** Baseline characteristics at initiation of rGH Therapy

Variable	Total *N* = 60	GHD *n* = 23	ISS *n* = 12	SGA *n* = 9	TS and variants *n* = 7	Others *n* = 9	*P* value
Female, n (%)	29 (48.3 %)	9 (39.1 %)	6 (50.0 %)	4 (44.4 %)	7 (100.0 %)	3 (33.3 %)	0.917*
Nationality, Kuwaitis n (%)	44 (73.3 %)	16 (69.6 %)	7 (58.3 %)	8 (88.9 %)	4 (57.1 %)	9 (100.0 %)	0.122
Age years, median (IQR)^♦^	9.0 (6.2, 10.7)	6.4 (5.5, 10.7)	10.6 (8.1, 10.9)	7.8 (7.2, 9.7)	9.9 (5.9, 10.7)	8.4 (5.0, 12.4)	0.407
Pre-pubertal, n/N (%)	50/59 (83.3 %)	21/23 (91.3 %)	10/12 (83.3 %)	9/9 (100.0 %)	5/7 (71.4 %)	5/8 (62.5 %)	0.127
Height SDS, mean (SD)^♣^	−2.77 (0.55)	−2.76 (0.50)	−2.84 (0.52)	−2.49 (0.54)	−2.82 (0.69)	−3.03 (0.69)	0.432
BMI SDS, median (IQR)^♠^	−0.46 (−1.25, 0.10)	−0.32 (−1.25, 0.27)	−0.44 (−1.91, −0.04)	−0.94 (−2.48, −.58)	0.10 (−0.31, 0.65)	−0.27 (−0.71, 0.24)	0.176

rGH: Recombinant Growth Hormone ; GHD: Growth Hormone Deficiency; ISS: Idiopathic Short Stature; SGA: Small for Gestational Age; TS: Turner Syndrome. SD: Standard Deviation; SDS: Standard Deviation Score; BMI: Body Mass Index; *test was done excluding TS. ^♦^Missing for 1 case (other indications); ^♣^Missing for 5 cases (2 TS and 3 other indications); ^♠^Missing for 5 cases (2 TS and 3 other indications).

A total of 44 patients had documented data on 1-year treatment response. Data on the characteristics of the patients and the parameters of the treatment response at 1-year follow-up are shown in Table 2. For all indications combined, 33 patients (75.0 %) had significant response to rGH (i.e. height SDS change ≥ 0.3). Children in all groups had a median height SDS change of ≥ 0.3 SDS following the first year of therapy except for children with ISS who had a median height SDS change of 0.17 (*p* = 0.005). Moreover, only 4 out of 10 children (40 %) with ISS had a significant response to rGH compared to other indications (*p* = 0.012). The median height SDS change observed ranged from a minimum of 0.17 in children with ISS to a maximum of 0.74 in children with SGA. At 1-year follow-up, there were no significant differences in puberty status and BMI change between the groups. No adverse effects were reported in the medical records except for one patient who complained of headache. Patients with no documented 1-year follow-up data had baseline characteristics similar to the rest of the study group [median (IQR) age 9.6(6.2–11.2) year; 8 are females].

**Table 2:** Characteristics of patients at 1-year follow-up with rGH Therapy

Variable	Total *n* = 44	GHD *n* = 18	ISS *n* = 10	SGA *n* = 9	TS and variants *n* = 4	Others *n* = 3	*P* value
Female, *n* (%)	21 (47.7 %)	7 (39.0 %)	5 (50.0 %)	4 (44.4 %)	4 (100.0 %)	1 (33.3 %)	0.279*
Age in years, median (IQR)	9.7 (7.2, 11.6)	7.3 (6.6, 11.6)	11.6 (9.7, 12.1)	8.9 (8.1, 10.7)	10.7 (8.6, 11.4)	7.7 (3.7, 14.7)	0.230
Pre-pubertal, n/N (%)	30/37 (81.1 %)	14/16 (87.5 %)	6/9 (66.7 %)	6/7 (85.7 %)	2/3 (66.7 %)	2/2 (100.0 %)	0.641
Height SDS, mean (SD)	−2.14 (0.59)	−2.11 (0.57)	−2.49 (0.55)	−1.82 (0.59)	−2.28 (0.73)	−1.98 (0.21)	0.158
Height SDS change, median (IQR)	0.54 (0.29, 0.75)	0.57 (0.33, 0.83)	0.17 (0.16, .41)	0.74 (0.59, 0.76)	0.40 (0.22, 0.61)	0.77 (0.44, 0.93)	0.005
BMI SDS, median (IQR)	−0.73 (−1.57, −0.21)	−0.73 (−1.34, −0.36)	−0.51 (−1.22, −0.09)	−1.59 (−2.00, −1.05)	−0.31 (−1.61, 1.02)	−0.19 (−0.59, 0.35)	0.136
BMI change, median (IQR)	−0.04 (−0.49, 0.24)	−0.23 (−0.49, 0.07)	0.19 (−0.34, 0.59)	0.17 (−0.59, 0.48)	−0.32 (−1.01, 0.43)	−0.36 (−0.43, 1.06)	0.431
Significant responders, n (%)	33 (75.0 %)	15 (83.3 %)	4 (40.0 %)	9 (100.0 %)	2 (50.0 %)	3 (100.0 %)	0.012

*rGH* Recombinant Growth Hormone, *GHD* Growth Hormone Deficiency *ISS* Idiopathic Short Stature, *SGA* Small for Gestational Age, *TS* Turner Syndrome. *SD* Standard Deviation, *SDS* Standard Deviation Score, *BMI* Body Mass Index. *test was done excluding TS.

Table 3 shows the association between baseline factors and significant response to rGH therapy using logistic regression. Age at rGH initiation was negatively associated with significant 1-year treatment response, Adjusted odds ratio (AOR) 0.56 (95 % CI: 0.36- 0.87; *p* = 0.011). Sub-group analysis to investigate the association between baseline factors and significant response to rGH in each group could not be performed due to small numbers. When the analysis was restricted to GHD or ISS, there was no significant association between the age at initiation of rGH therapy and significant response to the treatment, AOR for age at initiation 0.07 (95 % CI: 0.34 – 1.43, *p* = 0.326) and 0.22 (95 % CI: 0.02 – 2.76, *p* = 0.241), respectively.

**Table 3:** Association between baseline factors and significant response to rGH at 1-year follow-up in 44 patients

Baseline factors at rGH initiation	Odds Ratio [95 % CI]	*P* value
Male Gender	0.24 [0.04–1.49]	0.127
Age at initiation	0.56 [0.36–0.87]	0.011
Pre-pubertal at initiation of therapy	0.06 [0.001–1.98]	0.114
Height SDS at initiation	2.61 [0.41–16.80]	0.310
BMI SDS at initiation	0.49 [0.22–1.12]	0.092

*rGH* Recombinant Growth Hormone, *SDS* Standard Deviation Score, *BMI* Body Mass Index; *Height SDS change ≥0.3.

## Discussion

This study aimed to describe the pattern of use of rGH and the treatment outcomes. The study revealed that the most common indication for the use of rGH among children with short stature in Kuwait is GHD, which is similar to other reports from Europe and North America [4, 16]. However, different from patients reported in the literature, our patients’ population received rGH therapy at an earlier age for all indications (9.0 years) compared to the French SAGhE Study (11.0 years) [17]. Also, children with GHD in our study started rGH therapy at median age of 6.4 years compared to 10.2 years from the International ANSWER Registry [4], 10.8 years from USA [18], 9.2 years from the Pfizer International Growth Database (KIGS) [19] and 8.25 years from Taiwan [20]. For children with ISS, the age of initiation of therapy in a systematic review ranged from 6.1 to 12.9 years [21] compared to 10.6 years in our study. Another striking difference between our patients and patients described in the literature is the absence of gender difference in children receiving rGH in all indication groups combined or in each group separately. It has been reported elsewhere that males are more likely to receive rGH therapy compared to females [22, 23], which might be due to either parental gender preferences that affect seeking the treatment or differential referral. Equal numbers of males and females receiving rGH therapy in all indications suggest that parental gender preferences and differential referral are not strong in our setting. Nevertheless, it should be noted that in our study only 9 out of 23 patients with GHD were females. Although not statistically significant (*p* ≥ 0.40), influence of gender on referrals and seeking rGH therapy may warrant further investigation in our setting. Furthermore, gender differences in baseline height SDS were reported in the literature, where female subjects had lower mean height SDS compared to males across all indications groups which was not found in our patients’ population [18]. This gender difference has been also attributed to referral bias as male children are usually recognized to have short stature earlier than female children [24].

In our study, children with GHD were shorter (SDS mean −2.76) compared to patients reported from Europe and North America (i.e. The International ANSWER Registry SDS mean −2.3 [4], and USA −2.2 [18]) but were of similar heights to patients from Taiwan (SDS mean −2.78) [20]. On the other hand, those with ISS were shorter (SDS mean −2.84) than those reported in the International ANSWER Registry (SDS mean −2.3) [4], the French SAGhE Study (SDS mean - 2.7) [17] and USA (SDS mean −2.3) [18]. However, children with SGA were taller (SDS mean −2.49 ) than children in other studies such as the International ANSWER Registry (SDS mean −3.1) [4], USA (SDS mean −2.6) [18], and Denmark (SDS median −3.0) [25]. It is not clear if such differences reflect genuine variation in patients’ profile between our setting and other settings, and thus these differences should be interpreted cautiously because of the small numbers.

At 1-year follow-up, all groups had on average significant increment in height except for children with ISS with only 4 patients out of 10 having significant response to rGH therapy. It has been reported that the effectiveness of rGH for children with ISS is on average less than that achieved in other conditions for which the therapy is licensed [21]. This is why there is no unified consensus on the use of rGH for children with ISS. While the International Society of Pediatric Endocrinology and the Growth Hormone Research Society recommend rGH therapy for these children [11], the European Agency for Evaluation of Medicinal Products has not yet approved the use of rGH in children with ISS [9]. It has been suggested that the age at initiation of rGH therapy is negatively associated with the response to rGH therapy in various indications including ISS [26, 27]. The Consensus Statement on Diagnosis and Treatment of Children with ISS indicates that the optimal age for initiation of treatment is five years to early puberty [11]. It should be noted that children with ISS started receiving rGH therapy at a relatively older age in our setting. The median age of this group was 10.6 years, which makes this group older than any other treatment group in our setting (Table 1), and also near the upper limit of the recommended age range for rGH therapy (5 years to early puberty) [11]. Results from the meta-analysis of 10 trials with 1-year follow-up data on response of children with ISS to rGH therapy showed a mean change of height SDS of 0.43 which is higher compared to the response of children in our study [28]. This may indicate a different unknown etiology of short stature in children with ISS in our region. ISS is diagnosed when children with short stature have no evidence of systematic, endocrine, nutritional, or chromosomal abnormalities [29]. Therefore, ISS is not a single homogenous disease entity with a well-known underlying cause. It may include normal variants of growth, such as familial short stature and constitutional delay of growth and puberty, both characterized by achievement of an adult height within the range of mid-parental height [21]. These variants in growth have not been studied in children in Kuwait and might be of different nature compared to other children in different parts of the world. Our data highlight the need for further investigation of the treatment outcome of rGH in children with ISS in our setting.

It has been shown that total gain in height SDS correlates significantly with pre-pubertal gain in height SDS and younger age at start of treatment in GHD [4, 18, 26]. Therefore, the Growth Hormone Research Society advises that treatment should be initiated as soon as the diagnosis is made although no specific optimal time was specified for children diagnosed with GHD [1, 2, 30]. Early age at start of therapy was identified as a predictor of adult height in children with ISS as well [18, 21, 26, 27]. In the present analysis, it was found- as reported in the literature- that younger age at initiation of therapy is significantly associated with significant response to the treatment in all indications combined. However, due to small sample size, such relation was not statistically significant in children with GHD and ISS. Furthermore, gender was not significantly related to the response to rGH in the present study, which is consistent with larger studies investigating response to rGH therapy such as the KIGS database [31]. Anthropometric measures at initiation of therapy have been suggested to predict positive response to rGH; namely lower baseline height and higher BMI [32]. Such association was not demonstrated in our study, where baseline height and BMI were not significantly related to treatment response. This might reflects the different nature of growth in children in our region compared to other parts of the world.

Our study has several limitations including small number of patients and short period of follow-up. Because of this we were unable to detect meaningful differences. Our data also were obtained from one pediatric endocrine center, although a large center, but this might limit the generalizability of the results to other centers in the country and the region. The study was a cross-sectional retrospective review which relied on obtaining data from medical records. There was no data on several parameters (e.g. mid-parental height, target height, bone age, IGF-1 and IGF-BP3 levels, and GH doses at 1-year follow-up) which might influence response to rGH therapy. We have 16 patients with no follow-up data of whom eight have not reached 1-year of follow since the treatment. Their baseline characteristics are similar to the rest of the study group. Despite this, our study has several strengths. This study was conducted in one pediatric endocrine center which led to less variability in anthropometric measurements. Furthermore, it was an observational study of a real-life use of rGH rather than a controlled trial of therapy which cannot be ideally translated into everyday clinical practice.

## Conclusion

In this analysis, we have demonstrated that GHD is the most common indication of rGH therapy; and that there is minimal gender difference in receiving rGH therapy in Kuwait. Our patients seem to receive rGH therapy at a younger age (except for ISS) compared to other settings. Height SDS change at 1-year of rGH therapy showed significant response in all indications except for patients with ISS. rGH treatment outcomes in patients with ISS should be further investigated in Kuwait. Younger age at initiation of rGH therapy was independently associated with significant response to the treatment at 1-year follow-up. This suggests the importance of identifying children with short stature and prompt rGH treatment which is more affordable in Kuwait than in other settings in the Middle East. Future multi-center longitudinal study with adult height data is needed to investigate the treatment outcomes of rGH therapy in children in Kuwait and the region. In order to identify factors predicting response to therapy, the study should include parameters such as mid-parental height, target height, bone age, IGF-1 and IGF-BP3 levels, GH doses, and genetic studies. This is critical to guide decision makers to optimally use the resources for healthcare.

## Abbreviations

AOR: Adjusted odds ratio
BMI: Body mass index
GHD: Growth Hormone Deficiency
IGF-1: insulin-like growth factor-1
IGF-BP3: insulin –like growth factor – binding protein 3
ISS: Idiopathic short stature
rGH: Recombinant Growth Hormone
SDS: standard deviation score
SGA: small for gestational age
TS: Turner syndrome
